# Clinical Utility of the Cryptococcal Antigen Lateral Flow Assay in a Diagnostic Mycology Laboratory

**DOI:** 10.1371/journal.pone.0049541

**Published:** 2012-11-14

**Authors:** Brendan J. McMullan, Catriona Halliday, Tania C. Sorrell, David Judd, Sue Sleiman, Debbie Marriott, Tom Olma, Sharon C-A. Chen

**Affiliations:** 1 Centre for Infectious Diseases and Microbiology Laboratory Services, Westmead Hospital, Westmead, New South Wales, Australia; 2 Westmead Clinical School, University of Sydney, Westmead, New South Wales, Australia; 3 Sydney Emerging Infections and Biosecurity Institute, University of Sydney, Sydney, New South Wales, Australia; 4 Westmead Millennium Institute, Westmead, New South Wales, Australia; 5 Department of Microbiology and Infectious Diseases, St. Vincent's Hospital, Sydney, New South Wales, Australia; Charité, Campus Benjamin Franklin, Germany

## Abstract

**Background:**

*Cryptococcus neoformans* causes life-threatening meningitis. A recently introduced lateral flow immunoassay (LFA) to detect cryptococcal antigen (CRAG) is reportedly more rapid and convenient than standard latex agglutination (LA), but has not yet been evaluated in a diagnostic laboratory setting.

**Methods:**

One hundred and six serum, 42 cerebrospinal fluid (CSF), and 20 urine samples from 92 patients with known or suspected cryptococcosis were tested by LA and LFA, and titres were compared. Results were correlated with laboratory-confirmed cryptococcosis. Serial samples were tested in nine treated patients.

**Results:**

Twenty-five of 92 patients had confirmed cryptococcosis; all sera (n = 56) from these patients were positive by LFA (sensitivity 100%, 95% confidence interval (CI) 93.6–100%) compared with 51/56 positive by LA (sensitivity 91.1%, 95% CI 80.7–96.1%). Fifty sera from 67 patients without cryptococcosis tested negative in both assays. While LA yielded more false negative results (5/56) this did not reach statistical significance (p = 0.063). Nine CSF samples from patients with cryptococcal meningitis yielded positive results using both assays while 17/18 urine samples from patients with cryptococcosis were positive by the LFA. The LFA detected CRAG in *C. gattii* infection (n = 4 patients). Agreement between titres obtained by both methods (n = 38 samples) was imperfect; correlation between log-transformed titres (r) was 0.84. Turn-around-time was 20 minutes for the LFA and 2 h for LA. The cost per qualitative sample was 18USD and 91 USD, respectively and per quantitative sample was 38USD and 144USD, respectively.

**Conclusions:**

Qualitative agreement between the LFA and LA assays performed on serum and CSF was good but agreement between titres was imperfect. Ease of performance of the LFA and the capacity for testing urine suggest it has a role in the routine laboratory as a rapid diagnostic test or point-of-care test.

## Introduction

Cryptococcosis is a life-threatening infection caused by two main species, *Cryptococcus neoformans* and *Cryptococcus gattii*. Individuals with impaired cell-mediated immunity, especially those with HIV/AIDS and following organ transplantation, are at highest risk of infection but immunocompetent patients are also affected [1]–[4]. Worldwide, most cases of cryptococcosis are caused by *C. neoformans* (serotypes A, D and AD), predominantly in immunocompromised persons. In Australia, however, the incidence of infection in healthy hosts is high (31% of cases; overall incidence of 6.6 cases per million population/year) [4]. *Cryptococcus gattii* (serotypes B and C), which is endemic in Australia, causes disease predominantly in immunocompetent hosts (87% of cases) [4]–[6] *C. gattii* has also been reported as an emerging pathogen in British Columbia, Canada and in the United States [7], [8]. Meningitis is the commonest form of disease although primary respiratory illness is more common in Southeast Asia [1].

Despite appropriate antifungal therapy, mortality from cryptococcal meningitis (CM), the most severe form of cryptococcosis, remains high with death rates of 55–70% in HIV/AIDS patients in middle-to-low income countries and 15%–20% at 3 months in HIV-infected and non HIV-infected individuals in countries where HAART is available [1], [9]. Various strategies including early diagnosis and targeted screening have been proposed to reduce CM-related deaths. Cryptococcal polysaccharide antigen (CRAG) tests, most often in latex agglutination (LA) or enzyme immunoassay (EIA) formats, performed on serum or cerebrospinal fluid (CSF), are sensitive and specific methods for detection of CM [10]. These tests are also suitable for screening asymptomatic immunocompromised patients. This has significant clinical implications since, in otherwise asymptomatic HIV-infected persons, the presence of cryptococcal antigenemia predicts mortality [11]. Samples for LA or EIA CRAG assays must be refrigerated pending assay, and pre-processed by exposure to enzymes or heat. Test performance requires some technical expertise and interpretation of the endpoints can vary between operators.

In July 2011, the United States FDA approved a lateral flow assay (LFA; Immuno-Mycologics, Inc., OK, USA) for the rapid (≤15 mins) semi-quantitative detection of CRAG in serum or CSF [12]. The test consists of immune-chromatographic dipstick-like strips impregnated with monoclonal antibodies (Mabs) and optimized to detect all four major cryptococcal serotypes. Evaluation of the LFA against culture and EIA in Thai and African HIV-infected patients with/without CM found that the assay had sensitivities of 96–100% for serum and plasma, and 71–92% for urine, with test agreements of >93% [13], [14]. Unpublished data from a small number of HIV-infected Ugandan patients [15] suggest similar high sensitivity using LFA in CSF compared with LA; however larger data sets are required to confirm these observations. The utility of the LFA in HIV-negative individuals, *C. gattii* infections and in monitoring response to antifungal therapy has not been formally evaluated.

In the present study, we investigated the performance of the LFA for routine testing of samples within a hospital mycology laboratory using serum, CSF and opportunistic urine samples from the same patients. We compared the performance of the LFA and the LA test in the diagnosis of CM and other forms of cryptococcosis, in a setting where both *C. neoformans* and *C. gattii* are prevalent, and where many proven cases occur in non HIV-infected patients. We also assessed the performance of the LFA during antifungal therapy in a subset of patients. Finally, we investigated the practicality of replacing the CRAG LA test, routinely used in our diagnostic laboratory, with LFA.

## Materials and Methods

### Ethics Statement

Approval was obtained from the Human Research Ethics Committee of the Sydney West Area Health Service. As the study was performed using retrospective or existing samples with no intervention arm, the Ethics Committee waived the need for patient consent.

### Patients and clinical specimens

The study included patients from two university hospitals in Sydney, Australia. Ten archived serum specimens from six patients on which the CRAG LA test (Meridian Biosciences, Ohio, USA) had been performed were retrieved from the hospital microbiology laboratories and tested for CRAG using the LFA. These had been collected within the previous 12 months and stored at −70 degrees Celsius. Subsequently, between May 2011 and April 2012, patients with newly diagnosed cryptococcosis (within 2 weeks of diagnosis) or suspected cryptococcosis, were identified prospectively from the microbiology laboratory databases. Serum and CSF samples, if CSF had been collected, were tested in parallel by CRAG LA and LFA. Urine samples (where available, i.e. collected for microbial culture) were tested by LFA only. Patient electronic medical records were examined for confirmation of cryptococcosis, which was considered proven if the organism was detected by one or more of culture, histopathology or molecular tests. Clinical information on patients with cryptococcosis was also collected with regard to site of cryptococcal infection, type of specimen, whether samples were collected at diagnosis (pre-treatment) or follow-up and when post-diagnosis follow-up occurred.

### Test procedures

Latex agglutination (LA) testing was performed on serum and CSF specimens using the CALAS™ Cryptococcal antigen latex agglutination kit, according to the manufacturer's instructions [16]. All specimens with a positive result were tested up to a dilution of 1: 8192.

The LFA was performed using the IMMY Cryptococcal lateral flow assay (Immuno-Mycologics, Inc., OK, USA; ABACUS ALS, Australia), according to the manufacturer's instructions for serum and CSF and previous reports for urine testing [12], [13]. In summary, one drop of LFA specimen diluent was added to a disposable test tube then 40 µL of specimen was added to the tube and mixed together. Subsequently, a CRAG LFA test strip was inserted into the specimen and read at 1-min intervals from 1 to 10 min (the manufacturer's instructions specify 10 minutes). A single control line indicated a valid negative test and a control and test line indicated a valid positive test. Quantitative testing was also performed on serum and CSF specimens. This involved an initial dilution of 1∶5, followed by 1∶2 serial dilutions to 1∶2560. All results were also read at 1-min intervals from 1 to 10 min [12]. Urine, collected as a mid-stream sample in sterile containers, was tested undiluted. Cultures for *Cryptococcus* were processed according to standard laboratory methods [17], [18]. Molecular diagnosis of *C. neoformans* complex was by PCR amplification and DNA sequencing of the fungal internal transcribed spacer (ITS1) region [19]. Laboratory staff, as part of routine testing, performed LA and cultures and was blinded as to LFA results. LFA was performed either by CH or BM, who were incompletely blinded to LA qualitative and clinical results. As they were members of laboratory scientific and medical staff, respectively, full blinding was not possible. Both were, however, blinded to the LA titre at the time of LFA testing.

### Statistics

For the purpose of calculating sensitivity and specificity, cultures positive for *C. neoformans* or *C. gattii* or histopathology or molecular testing consistent with *Cryptococcus* were considered positive for comparison with both LA and LFA. Patients considered “negative” were those with negative cultures and/or a proven alternate diagnosis and no evidence of development of cryptococcosis during the study period. Sensitivity and specificity were calculated using the Wilson method [20]. Agreement between results of the LA and LFA was quantified using correlation and a Bland-Altman plot. The Bland-Altman plot graphically displays agreement between two methods of measurement by plotting the differences between the two methods against their averages [21]. McNemar's test was used to compare the differences between proportions of qualitative results (positive or negative) obtained by both assays.

## Results

### Patient characteristics

Characteristics of 92 patients tested are shown in Table 1, including site of disease, immune status and method of diagnosis. Twenty-five patients were diagnosed with cryptococcosis and 67 had no evidence of cryptococcosis. The patients without cryptococcosis had variety of alternative diagnoses, including cerebral and pulmonary malignancies, meningitis and encephalitis due to other causes.

**Table 1:** Characteristics of patients and samples.

***Patient characteristics N = 92***	
Male sex no/(%total)	59 (64.1%)
Age range (median)	21–81 (47)
Cryptococcosis (%total)	25 (27.2%)
***Patients with cryptococcosis N = 25***	
**Site of disease: no/(%total)**	
Central nervous system	14 (56%)
Pulmonary	6 (24%)
Other*	5 (20%)
**Immune status: no/(%total)**	
Immunocompetent	12 (48%)
HIV-infected	4 (16%)
Other immunocompromised	9 (36%)
**Method of Laboratory Diagnosis: no/(%total)**	
*C. neoformans* culture positive	16 (64%)
*C. gattii* culture positive	4 (16%)
Histological diagnosis of *Cryptococcus* †	3 (12%)
Molecular diagnosis of *Cryptococcus*	2 (8%)
***Sample characteristics N = 168***	
Serum no/(%total)	106 (63.1%)
Cerebrospinal fluid no/(%total)	42 (25.0%)
Urine no/(%total)	20 (11.9%)

* Three patients had had fungemia and one each had laryngitis and osteomyelitis.

† Two patients had encapsulated yeast seen on Periodic acid-Schiff/mucicarmine staining. One patient had granulomatous inflammation seen on fine-needle aspirate but fungal stains were not performed, both lateral flow assay and latex agglutination were positive for this patient.

### Clinical specimens

A total of 168 samples from 92 patients were tested by the LFA and LA. These are listed by sample type in Table 1. Ten samples were stored sera (as described above) and the remaining 158 sera, CSF and urine specimens were samples tested prospectively in parallel with LA. For nine patients, serum and/or CSF and/or urine were available for testing by both LA and LFA at diagnosis of cryptococcosis, and during the course of antifungal therapy.

### Performance of LFA on serum

**Figure 1:**
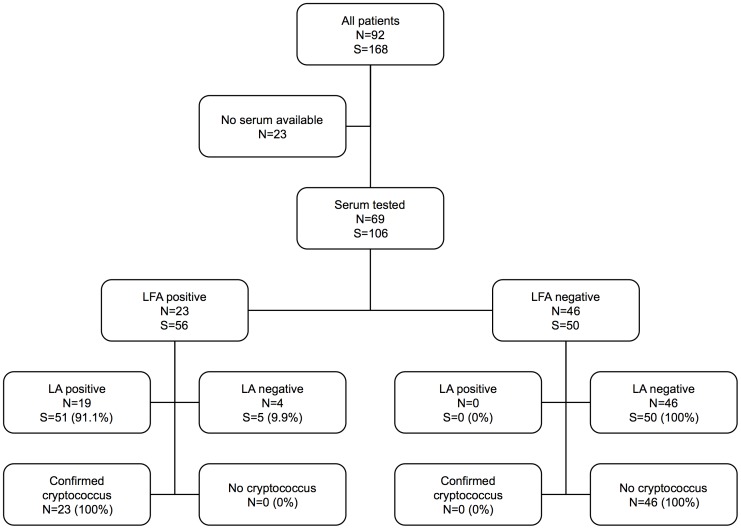
Serum Cryptococcal Antigen Results by Assay. Flow chart of detection of serum cryptococcal antigen for 92 patients by lateral flow assay (LFA) and latex agglutination (LA). (S) specimens, (N) patients.

A flowchart of results from serum testing is displayed in Figure 1. Fifty-six sera from patients with confirmed cryptococcosis were all positive by LFA (sensitivity 100%, 95% confidence interval 93.6–100%), compared with 51/56 positive by LA (sensitivity 91.1%, 95% confidence interval 80.7–96.1%). Fifty sera from patients without cryptococcosis yielded negative results by both LFA and LA (specificity 92.9–100% for both assays). While LA yielded more false negative results (5/56) this did not reach statistical significance (McNemar's test p = 0.063).

### Performance of the LFA on specimens other than serum

Nine CSF samples from patients with cryptococcal meningitis were positive by both the LFA and LA, and all 31 samples from patients without cryptococcosis were negative in both assays. Two CSF samples from a patient with isolated pulmonary cryptococcosis were negative in both assays. Seventeen of 18 urine samples from patients with confirmed cryptococcosis were positive by LFA (sensitivity 94.4%, 95% confidence interval 74.2–99%). The negative urine sample was collected from a patient with laryngeal cryptococcosis. *C. neoformans* was cultured from a biopsy of laryngeal tissue and the patient had a negative LA result on serum but positive qualitative LFA result (titre <5). Two urine samples from patients without cryptococcosis were negative by LFA.

### Performance of the LFA and LA on follow-up specimens

Clinical specimens (serum, CSF and urine) collected at diagnosis and at clinical follow-up (median 277 days, range 17–537 days post diagnosis) were available from nine patients. In general qualitative results for both assays were concordant for treated patients (data not shown). For one patient, however, who was treated for cryptococcal meningitis, CRAG was detectable by LFA for substantially longer than by LA. Both assays were positive at 160 days post diagnosis but at 219 and 421 days, CRAG was not detectable by LA but remained detectable by LFA at titres of 40 and 10, respectively.

### Agreement between LFA and LA titres

**Figure 2:**
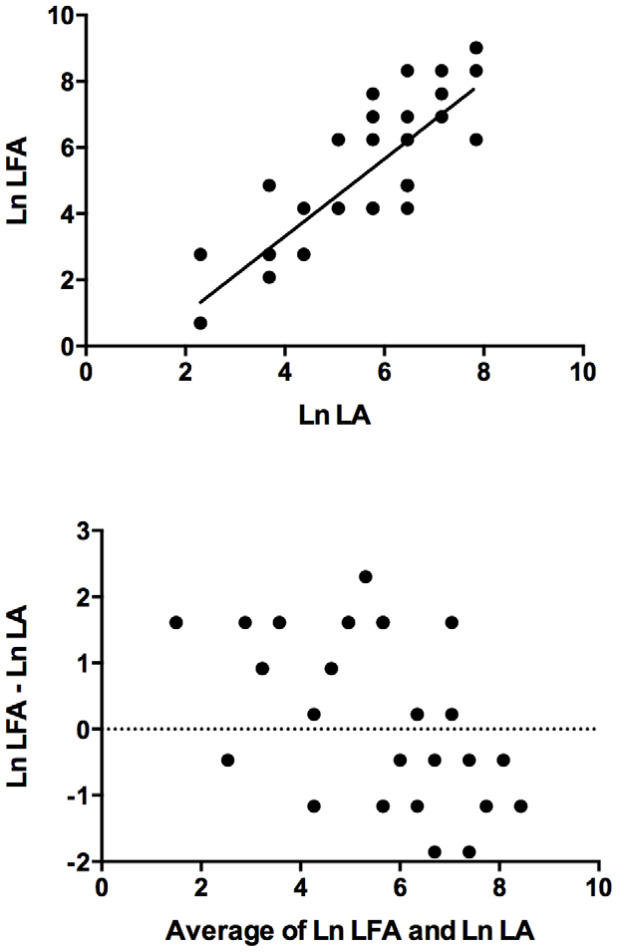
Agreement between Assays. Correlation (above) and Bland-Altman plot (below) for 38 samples tested by lateral flow assay (LFA) and latex agglutination (LA). Ln = log_e_.

Sufficient sample was available for quantitative testing by LFA and LA on 38 specimens (33 sera and five CSF samples). Correlation between log-transformed titres (r) was 0.84. Agreement between titres was imperfect: in general, LFA titres were higher than those obtained by LA (LFA: LA = 1.53), however confidence limits ranged from 0.13 to 18.1. These results are displayed graphically in log-transformed format in Figure 2.

### Utility of LFA as a point-of-care test (POCT)

The turn-around-time was 20 minutes for qualitative or quantitative LFA and 2 h for quantitative LA testing. Cost per sample tested quantitatively was 38USD by LFA compared with 144USD by LA. Cost per sample tested qualitatively only was 18USD per sample by LFA or 91USD by LA. These costs are based on testing a single sample and include the cost of technical officer time. Costs for additional samples tested in batches were 2USD or 4USD per sample for LFA and LA, respectively. Investigators were able to perform LFA testing confidently and accurately with reference to package insert instructions after a single demonstration. Although samples were examined for up to 10 minutes by LFA, all positive LFA results were visible within 6 minutes.

## Discussion

In this study, we determined the clinical utility of the LFA for the diagnosis of cryptococcosis in a diagnostic microbiology laboratory and assessed its potential as: (i) a replacement test for LA or EIA CRAG detection; and (ii) an after-hours point of care test for the rapid diagnosis of cryptococcosis. This question has relevance in both high-, and low-income settings. To date, the use of LFA for the diagnosis of cryptococcosis has been evaluated under research laboratory conditions only and compared primarily to EIA, rather than the widely used LA that we examined in our study. Our study included 25 patients with a variety of clinical manifestations of cryptococcosis, immunocompromised (both due to HIV and other causes) and immunocompetent patients, reflecting the spectrum that our diagnostic laboratory encounters in practice. Four patients in our study had culture-confirmed *C. gattii* disease, for which evaluation of the LFA has not been published. The 67 patients without cryptococcosis in our study had a range of conditions, the differential diagnosis of which included cryptococcosis. We have demonstrated that, in a routine laboratory setting, the LFA test is rapid, sensitive, specific and of lower cost than LA.

The LFA assay was simple to use with minimal training. In addition, as it does not require heat or enzyme treatment, strips can be stored at room temperature and it is suitable for use on serum, plasma and urine. The LFA also offers advantages as a POCT for the diagnosis of cryptococcosis and as a screening tool in HIV-infected individuals. The World Health Organisation WHO has stated that the LFA largely meets their ASSURED criteria for POCT (Affordable, Sensitive, Specific, User-Friendly, Rapid, Equipment-free, and Delivered to those who need it). Furthermore, WHO has recommended that the LFA be used to screen patients with HIV infection [22]. Notably, CRAG is detectable in peripheral blood prior to the onset of symptoms of CM by an average of 22 days and approximately 11% of people will have antigen present 100 days before disease onset [15]. The LFA's ability to be performed urine samples has clear advantages for testing in remote settings or where invasive samples may be impractical to obtain.

Qualitative agreement between LFA and LA performed on serum and CSF was very good. Discrepancies between the two tests were due to a small proportion of false-negative LA results, rather than false-positive LFA results, suggesting the LFA is a more sensitive assay, though, in our evaluation, differences did not attain statistical significance. This may be important in detecting patients with pre-clinical or early disease who may have a low antigen burden.

Quantification of CRAG levels by LFA on serially diluted samples requires further evaluation, in particular, in comparison with those obtained by LA and EIA, to validate use of this method as a prognostic indicator in cryptococcal meningitis. Serum or CSF titres greater than 512, when tested by LA or EIA, have been correlated with mycological failure at two weeks [23] and high serum or CSF titres during therapy have been associated with relapse in HIV-infected patients [9]. Based on unpublished data from a Ugandan cohort, cited in a recent review [15], it was suggested that the ratio of titres measured by latex agglutination versus LFA is a consistent 1∶5. Our evaluation indicates that agreement between LFA and LA is imperfect. Although the correlation coefficient (r) between log-transformed titres was 0.84, the ratio of LFA to LA (actual titres) was 1.53, with wide confidence limits. We chose to display correlation and agreement graphically using a scatter plot and a Bland-Altman plot, respectively (Figure 2). As Bland and Altman have demonstrated [21], although correlation is often reported as a measure of agreement, in fact the correlation coefficient (r) measures the strength of a relation between two variables, not the agreement between them. In addition, correlation improves with the range of the true quantity (level of a substance) in the samples tested. For these reasons, even a high correlation between two test methods does not necessarily indicate good agreement between the actual values obtained. Thus simple reporting of a correlation coefficient and/or scatter diagram to demonstrate agreement, although common, may be misleading and is not appropriate in isolation for comparing agreement between two test methods. The interpretation of LA test results is also operator-dependent and in a routine laboratory different staff will perform these assays. This may have contributed to the imperfect agreement of titres obtained by LA and LFA in our study.

We suggest that titres obtained by LFA should not be translated directly into equivalent LA or EIA titres for use as prognostic determinants or to monitor response to treatment in individual patients until further evaluation with larger representative samples is performed. A practical implication of this for the clinical laboratory is that titres obtained in a single patient by LFA cannot be compared directly with those obtained using LA or EIA. This may include patients being monitored on treatment or post-treatment for cryptococcosis. Laboratories may consider keeping some LA test kits for these patients or testing with LA and LFA in parallel (if phasing LA out) and provide a comment indicating by which method titres were obtained.

Potential limitations of our study include the relatively small sample size and the fact that for most patients, clinical data were retrieved from electronic medical records, rather than recorded in real time by clinical research staff or the study authors. A number of patients resided in rural areas and were not reviewed personally by the investigators. Blinding was incomplete with respect to qualitative LFA testing as the investigators concerned worked in the same clinical hospital laboratory where routine LA testing and culture is performed. In fact, blinding was present in the majority of our cases, was complete in regard to quantitative (titre) results and LA testing, and is thus unlikely to have significantly influenced our results. It is noteworthy that, of the two largest published evaluations of CRAG LFA to date, neither was reported to be blinded [13], [14].

We conclude that the LFA is a promising diagnostic test for use in microbiology laboratories and as a POCT elsewhere. Further comparison of titres obtained by LFA and LA is required before it can be recommended that the LFA replace standard latex agglutination or EIA testing for epidemiological or prognostic purposes
